# Trajectory-based training enables protein simulations with accurate folding and Boltzmann ensembles in cpu-hours

**DOI:** 10.1371/journal.pcbi.1006578

**Published:** 2018-12-27

**Authors:** John M. Jumper, Nabil F. Faruk, Karl F. Freed, Tobin R. Sosnick

**Affiliations:** 1 Department of Biochemistry and Molecular Biology, University of Chicago, Chicago, Illinois, USA; 2 Department of Chemistry, and The James Franck Institute, University of Chicago, Chicago, Illinois, USA; 3 Graduate Program in Biophysical Sciences, University of Chicago, Chicago, Illinois, USA; 4 Institute for Biophysical Dynamics, University of Chicago, Chicago, Illinois, USA; Fox Chase Cancer Center, UNITED STATES

## Abstract

An ongoing challenge in protein chemistry is to identify the underlying interaction energies that capture protein dynamics. The traditional trade-off in biomolecular simulation between accuracy and computational efficiency is predicated on the assumption that detailed force fields are typically well-parameterized, obtaining a significant fraction of possible accuracy. We re-examine this trade-off in the more realistic regime in which parameterization is a greater source of error than the level of detail in the force field. To address parameterization of coarse-grained force fields, we use the contrastive divergence technique from machine learning to train from simulations of 450 proteins. In our procedure, the computational efficiency of the model enables high accuracy through the precise tuning of the Boltzmann ensemble. This method is applied to our recently developed *Upside* model, where the free energy for side chains is rapidly calculated at every time-step, allowing for a smooth energy landscape without steric rattling of the side chains. After this contrastive divergence training, the model is able to *de novo* fold proteins up to 100 residues on a single core in days. This improved *Upside* model provides a starting point both for investigation of folding dynamics and as an inexpensive Bayesian prior for protein physics that can be integrated with additional experimental or bioinformatic data.

## Author Summary

All-atom biomolecular simulations are useful but often take months to simulate biologically relevant reactions. Coarse-grain folding simulations reduce the computational requirements; however, they typically have reduced accuracy and knowledge of the native state is required. Here, we show that a properly formulated coarse-grain model trained using modern machine learning methods can rival all-atom models for *de novo* protein folding and dynamics simulations. The *Upside* model’s success argues that simpler models that can be globally parameterized can rival more detailed but slower models whose parameterization is more challenging—more complexity does not necessarily equate to higher accuracy. *Upside*’s ready generation of Boltzmann ensembles allows for a wide range of computational studies of protein folding, dynamics and binding. Additionally, in studies that incorporate experimental or bioinformatics data, including sparse contact predictions, *Upside* provides an inexpensive Bayesian prior distribution over protein structures that may be updated using experimental information.

## Introduction

Since Anfinsen’s original demonstration that a protein’s sequence determines its structure, multiple computational strategies have been developed to predict a protein’s structure from its sequence. An additional facet of this challenge is to replicate the energy landscape that defines both the folding process and other dynamical properties. In the absence of other information, coarse-grained models with one or a few beads per residue are too simplistic for *de novo* structure prediction. C_*β*_ level models having authentic protein backbones with *ϕ*/*ψ* dihedral angles, but lacking side chain rotamers, have achieved some success [1–3]. Within the last decade, all-atom, explicit solvent methods have become successful for the folding of some small proteins, although the ability to replicate the properties outside the native basin requires substantial improvement [4]. For the folding process, it is unclear which representation provides the optimal combination of detail and computational expense to replicate protein folding and dynamics. Integral to the choice of representation is which interactions to include, such as hydrogen bonding, van der Waals interactions and hydrophobic burial.

Another factor is the parameterization of the energy function with the training algorithm needing to balance the influences of all interactions. Protein thermodynamics reflects a delicate balance between the free energy of the folded and unfolded states. If one interaction is slightly too large, the entire landscape can be severely distorted. For example, if backbone hydrogen bonding energies are too large compared to backbone-solvent interactions (which includes hydrogen bonds between the backbone and water), an excess of hydrogen bonding ensues and pathways become dominated by unrealistically stable native- and non-native secondary structures. In an extreme situation, the lowest energy structure may have long helices involving nearly all residues.

The balancing of these various energies has been a major effort, and the balance is continually being adjusted as new force field biases are identified [5]. However, the adjustment of some parameters to correct one deficiency can inadvertently degrade performance of other quantities. In order to achieve the correct balance, all terms in the model should be trained together, rather than adjusted with an *ad hoc* procedure to correct each identified deficit.

To achieve this balance with a detailed interaction model, we use our recently developed, extremely rapid *Upside* implicit solvent molecular dynamics program [6]. Each residue In *Upside* is represented with a polypeptide backbone and a side chain interaction site or bead which can adopt up to 6 positions representing up to six different side chain *χ*_1_/*χ*_2_ states. The key advance of the model is the smoothing of the energy surface by approximate analytic integration of free energies for the side chains’ discrete states. When trained to predict side chain conformations from the Protein Data Bank (PDB), the method can fold a few small proteins with moderate accuracy in a cpu core-day. The majority of speedup of the procedure is a result of a unique side chain algorithm which directly calculates the side chain probability distribution and the free energy. This free energy calculation, performed at every time step, avoids the steric rattling of the side chains which can occur in the condensed phase in all-atom simulations, and so allows the backbone to move on a smoother energy landscape.

Here, we demonstrate that we can achieve *de novo* folding for a diverse collection of proteins by combining our fast-equilibrating *Upside* model with a contrastive divergence procedure that optimizes the stability of the native well. We demonstrate that gradient descent on energy terms using only data from sampled trajectories is sufficient to parameterize a protein model with tens of thousands of parameters. The resulting parameters are sufficiently balanced and accurate to achieve reversible folding for many proteins in our validation set. In addition, the resulting model is an excellent starting point for large scale protein simulations using more detailed models as well as the integration of large quantities of external information (such as predictions of residue contacts).

## Methods

### Coarse-grained model

**Fig 1:**
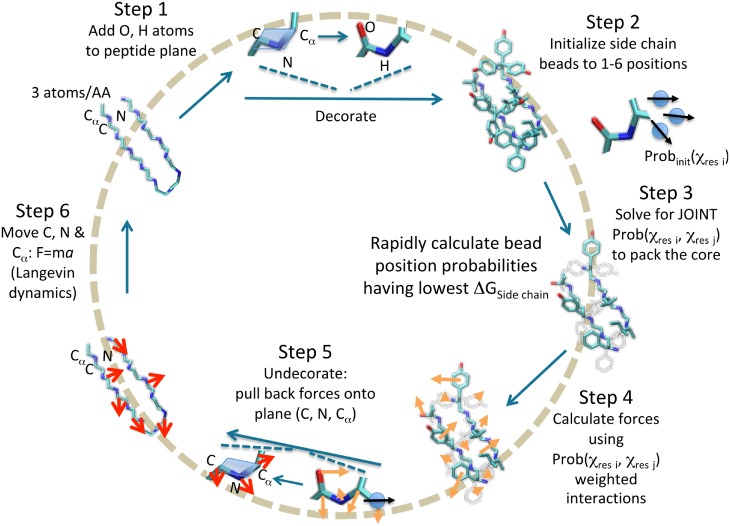
Computational inner loop for *Upside*. The positions of the protein side chains are added during each energy or force computation, then an approximate Boltzmann distribution is estimated for the side chains, and the free energy of the side chains is computed using the approximate Boltzmann ensemble. The resulting energy derivatives are pulled back to the backbone coordinates to update the backbone momenta.

In our recently-developed *Upside* model, only the N, C_*α*_, and C atoms for each residue undergo dynamics. This simple representation of the protein allows for molecular dynamics on a smooth landscape but also makes it challenging to include the entirety of the protein physics. To address this challenge, we build additional layers of derived coordinates during the energy computation, much like virtual sites in a traditional force field. These layers include amide hydrogens, carbonyl oxygens, hydrogen bonding and residue burial scores, and the possible locations of protein side chains. All of the derivative information required is backpropagated through these layers of representation during the computation of forces for molecular dynamics. The side chain positions are the most challenging to represent because we must solve a side chain packing problem in order to determine the distribution of side chain positions for a given backbone geometry. To pack the side chains probabilistically and obtain a side chain free energy, we use a rapid self-consistent iteration as described in our recent work [6] (Fig 1). The major computational steps are:

Step 1 The loop begins (upper left corner) with each residue in the protein being represented with 3 backbone atoms, the N, C_*α*_ and C. Based on the position of these atoms, the carbonyl oxygen, O, and amide proton, H, are deterministically placed.
Step 2 Each side chain, represented by a single oriented bead, is assigned an initial probability for being in 1–6 states, depending on the residue type and the average frequency observed in the PDB. The state of the bead is defined by its position and an orientation, (x,y,z,v), where v is a unit vector, relative to the peptide plane.
Step 3 The pair-wise state probabilities of all side chains are simultaneously and rapidly calculated using belief propagation to produce the lowest system free energy.
Step 4 Forces on the 3 backbone atoms, as well as on the O, H and side chain beads are calculated from the derivative of the free energy.
Step 5 Forces on the O, H and bead are “pulled back” and added to the forces on the 3 backbone atoms by reversing the placement process.
Step 6 Langevin dynamics (implicit solvent with friction) are run on the 3 backbone atoms using the forces calculated in Steps 4 and 5.

The majority of parameters in *Upside* define the pairwise interactions between side chains, where each side chain is represented by a single directional bead. Concretely, each interaction pair is described by bead positions *y*_1_ and *y*_2_ and their orientations *n*_1_ and *n*_2_. From the distance *r*_12_ = |*y*_1_ − *y*_2_| and displacement unit vector *n*_12_ = (*y*_1_ − *y*_2_)/*r*_12_ are calculated. All of the pairwise interactions have the functional form

$$\begin{array}{cc} V= & \kappa (V_{\text{radial}}\left(r_{12}\right)+\\ & \;{\text{ang}}_{1}\left(-n_{1}\cdot n_{12}\right)\;{\text{ang}}_{2}\left(n_{2}\cdot n_{12}\right)V_{\text{angular}}\left(r_{12}\right)), \end{array}$$ (1)

where *V*_radial_, ang_1_, ang_2_, and *V*_angular_ are smooth curves represented by cubic splines for increased flexibility, rather than fixed functional forms such as a van der Waals 6-12 potential. The potential for each of the (202)+20=210 types of amino acid pairs are described with 62 spline coefficients per pair, giving 13020 parameters. There are also five interaction sites on the backbone, roughly representing the H, O, N, C_*α*_, and C atoms, with 54 parameters per interaction due to a smaller cutoff distance (10 versus 8 Å). The total number of side chain-backbone interaction parameters is 5400.

We add an additional term to capture desolvation effects by computing the approximate number of side chains *N*_*i*_ within a hemisphere above the C_*β*_ (see S1 Text in Supporting Information). High values of *N*_*i*_ correspond to buried residues. The total energy is

$$\begin{array}{c} V_{\text{env}}=\sum_{i}v_{a_{i}}^{\text{env}}\left(N_{i}\right), \end{array}$$ (2)

which is the sum of the values from individual $v_{a_{i}}^{\text{env}}$ potential curves for each residue *i*. Although more sophisticated solvation potentials exist, our implementation is very fast and easily optimized by the contrastive divergence procedure, while remaining flexible enough to represent many of the solvation effects omitted by the pairwise side chain potential.

The backbone dihedral angle Ramachandran potential is $\sum_{i}V_{i}^{\text{Rama}}\left(\phi_{i},\psi_{i}\right)$, where $V_{i}^{\text{Rama}}$ depends on the chemical identity of the *i* − 1, *i*, and *i* + 1 residues. The Ramachandran potentials are based on the turn, coil, or bridge (TCB) Ramachandran probability models in the NDRD backbone library [7]. We introduce a single parameter controlling extra stabilization of angles consistent with *β*-sheet geometries to allow training to counteract an observed tendency for our model to overstabilize helices. The backbone non-bonded interactions are governed by a distance- and angle-dependent hydrogen bonding potential whose magnitude (but not geometry) is chosen by contrastive divergence. The backbone N, C_*α*_, C_*β*_, and C feel a steric repulsive interaction when their internuclear distance is approximately 3.0 Å.

Source code for *Upside* can be obtained from https://github.com/sosnicklab/upside-md, and the results of this paper can be reproduced using the version tagged trajectory_training_paper.

### Contrastive divergence

**Fig 2:**
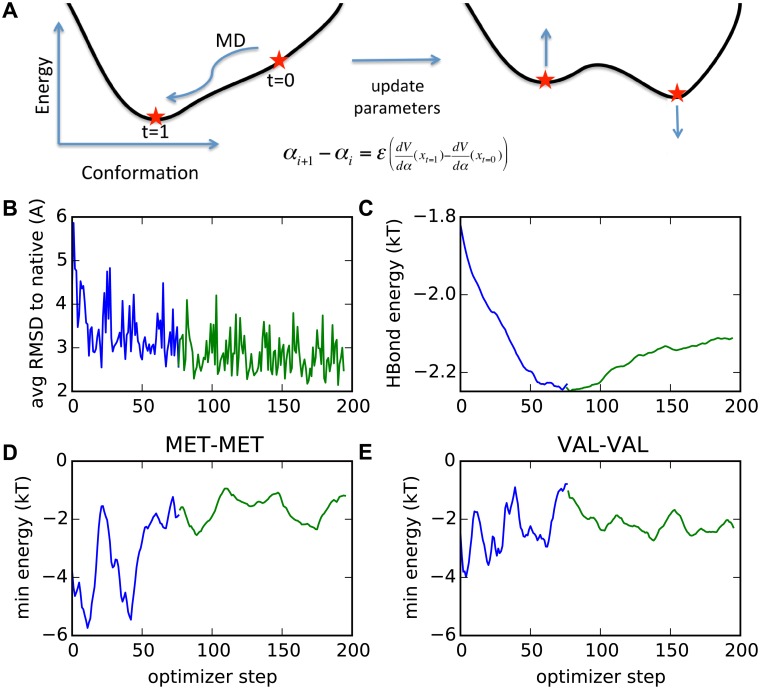
Contrastive divergence training. (A) Schematic of the training procedure depicting how the native state is stabilized relative to other states upon parameter updates. (B)-(E) In all plots, the blue curves indicate larger initial step-size training and the green plots indicate smaller step-size (fine-tuning). (B) The upper left plot shows the decline in minibatch-averaged RMSD over the course of the optimization. The remaining plots show (C) the convergence of the hydrogen bonding and side chain-side chain interaction parameters over the optimization for (D) Met-Met and (E) Val-Val potential. The larger step-size optimization of the side chain parameters exhibits large oscillations that inhibit convergence.

Our implementation of contrastive divergence considers two ensembles, one closely restrained to the native (crystal) structure and another that is free to diffuse away during simulations (Fig 2). In a perfect model, an unrestrained ensemble would remain close to the native structure. For an inexact model, differences arise, such as an excess of backbone-backbone hydrogen bonding in the free ensemble. Reducing the hydrogen bond energy would shift the free ensemble closer to the native ensemble. The parameter modification must be small, however, because shifting the hydrogen bond energy may adversely affect other features of the ensemble, e.g., by reducing the burial of hydrophobic residues. Accordingly, after simulations are run on the first set or “minibatch” of 12 proteins in our 456 protein training set, we modify all the parameters with small updates to shift the simulation ensemble to better match the native-restrained ensemble. Simulations are repeated on the next of the 38 subsets of 12 proteins, and the paramters are updated again. The algorithm is converged when no parameter can be altered to shift the free ensemble closer to the native-restrained ensemble.

The free ensemble is generated using 5000 time units of dynamics (approximately 10 wall-clock minutes), with the first half being discarded as equilibration. Unless the native state is particularly unstable, this time is insufficient for exploration of the conformational landscape much beyond the native basin (RMSD within 6 Å) and so produces only a locally-equilibrated ensemble.

The native ensemble is traditionally defined as a single conformation. This *δ*-function distribution is problematic for proteins because they are dynamical molecules. Additionally, the solution ensemble may differ from the crystal structure for multiple reasons, including crystallographic packing. To reduce the impact of these issues, we replace the exact ensemble structures with the ensemble restrained to be near the crystal structure, within approximately 1 Å C_*α*_-RMSD. This procedure is analogous to the restrained equilibration of crystal structures required to prepare systems for all-atom molecular dynamics. To account for changing parameters, we apply the restrained relaxation at every optimizer step.

After generation of the free and native-restrained ensembles, we change the energy parameters *α*_*i*_, where *i* is the optimizer step, in proportion to the amount that the change can differentiate the two ensembles. This procedure is a form of gradient descent to reduce the “distance” between the free and native-restrained ensembles,

$$\begin{array}{c} \alpha_{i+1}=\alpha_{i}+\frac{\epsilon }{M}\sum_{a=1}^{M}(\langle \frac{dV}{d\alpha_{i}}\rangle_{\text{restrained}}-\langle \frac{dV}{d\alpha_{i}}\rangle_{\text{free}}), \end{array}$$ (3)

where *ϵ* the step size, *M* is the number of proteins, and *a* indexes the simulated proteins. The quantity $\langle \frac{dV}{d\alpha_{i}}\rangle_{\text{restrained}}-\langle \frac{dV}{d\alpha_{i}}\rangle_{\text{free}}$ represents a pseudo-derivative of the free energy of restraining the simulation to be near the crystal structure (see SI for details). In the limit that the simulation duration is infinite, this difference is the exact derivative of the free energy. In practice, this difference chooses a suitable direction to improve the parameters.

The simulations use temperature replica exchange with eight replicas to enhance barrier crossing [8], while the temperature intervals of the replicas scale with $1/\sqrt{N_{\text{res}}}$ to encourage efficient replica exchange for proteins of various sizes. The progress of the replica exchange is monitored by the average RMSD-to-crystal structure over the simulation for each “minibatch”, the 12 protein subset used for a single gradient-descent step.

## Results

### Training

The parameters are initially set to those used to optimize side chain (*χ*_1_) accuracy [6]. The contrastive divergence training rapidly improves the model’s average RMSD over a minibatch from 6 Å to 3 Å. This decline is accompanied by rapid change in the parameters. To reduce parameter fluctuations and fine-tune the results, we reduce the optimizer step size by a factor of four after two full passes through the 38 minibatches.

Although the slope has greatly decreased of RMSD change with respect to the number of steps over the iterations, there are indications that the parameters have not yet converged. Earlier tests, however, showed that continuing the contrastive divergence until convergence does not necessarily produce better results, as has been previously observed [9]. When large barriers surround the native states, minimal relaxation of the conformation occurs, which in turn provides little new information, and further fine-tuning may even *reduce* the accuracy of the model. Potentially the decreased exploration in the native well in the later stages overtrains the model to distinguish between native and near-native structure at the expense distinguishing against a more diverse ensemble. Early termination of optimization has been observed to favor simpler models [10].

**Fig 3:**
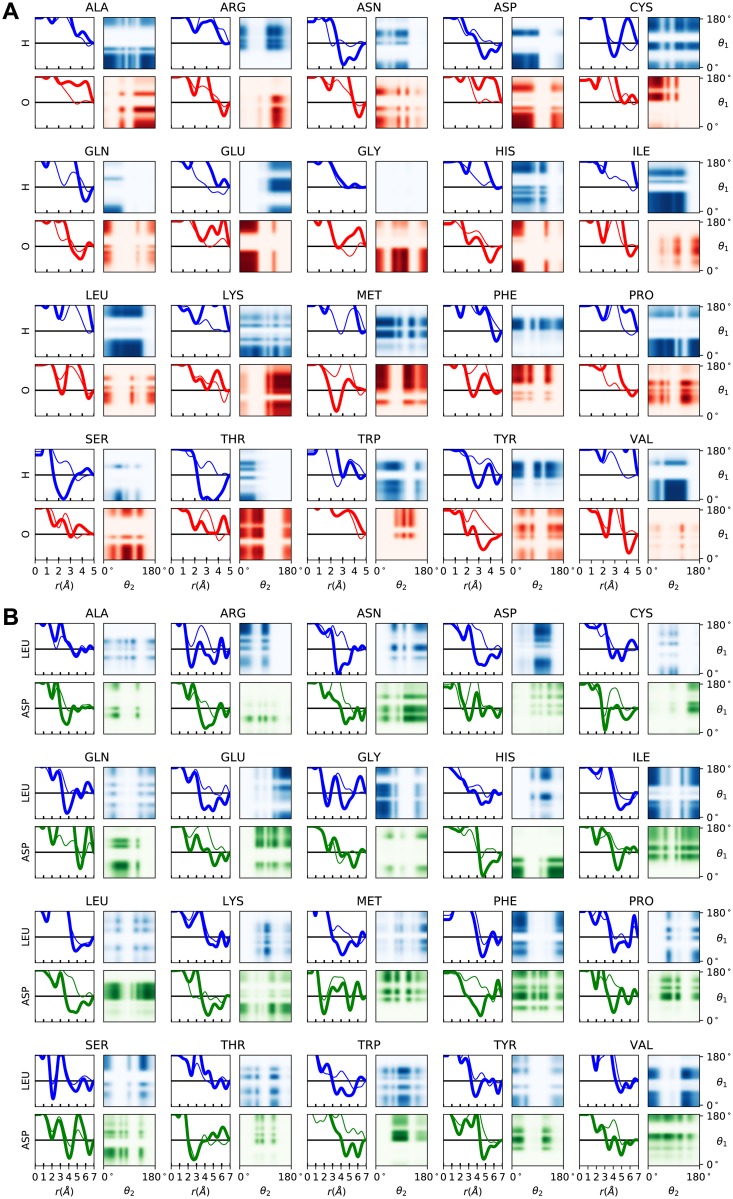
Representative pair interaction potentials from the contrastive divergence training. (A) Side chain bead-to-carbonyl oxygen and bead-to-amide proton (blue/red) and (B) side chain bead-to-bead (blue/green). Thin lines indicate *V*_radial_(*r*) while thick lines indicate *V*_radial_(*r*) + *V*_angular_(*r*) with a plot range of (−6kT, 6kT). The heat maps show the angular product ang_1_(*θ*_1_) ang_2_(*θ*_2_).

The hydrogen bond strength unexpectedly appears to converge to a significantly smaller value during the late, fine-tuning stage than during the early phase with larger optimizer steps. We speculate that the extra noise in the side chain interactions during the larger optimizer steps may in aggregate cause stronger side chain interactions for the protein. This effect would necessitate a large hydrogen bond energy to balance the increase in side chain interactions. The final pair-wise energy functions between the side chain beads and either the backbone carbonyl oxygen or the amide proton, and the bead-bead interactions are shown in Fig 3.

### Accuracy of structure prediction

**Fig 4:**
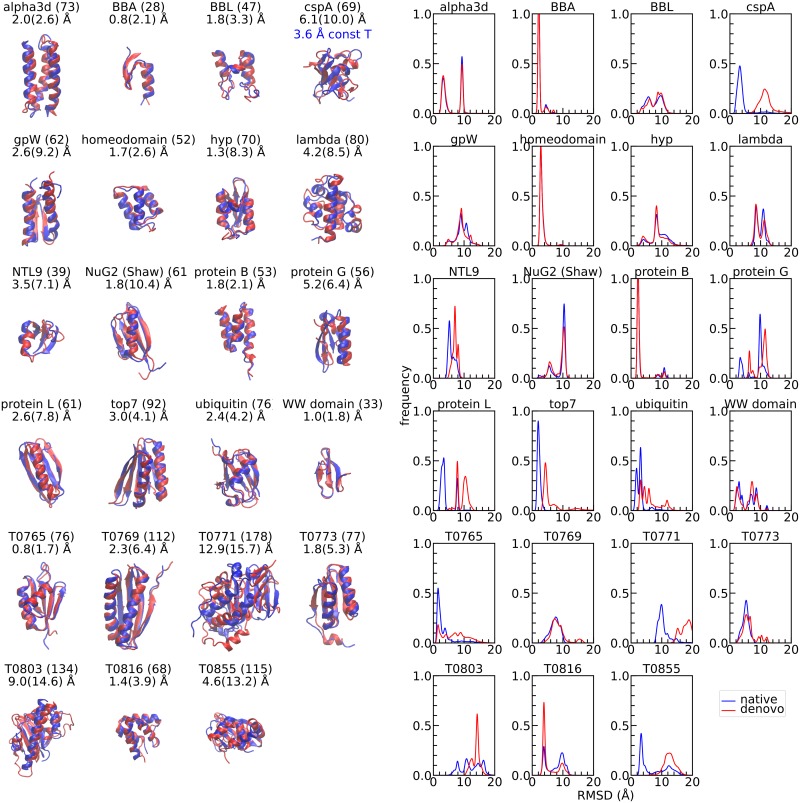
Predicted structures and C_*α*_-RMSD distributions. After equilibration phase for the lowest temperature of replica exchange simulations (see S1 Text). The simulations start from either the native (blue) or a random unfolded state (red). For the refolding simulations, the lowest C_*α*_-RMSD to native structures is provided along with the value for the centroid of largest cluster (in parentheses). RMSD calculations exclude three residues at the amino- and carboxy-termini to account for possible disorder at the ends. Each replica is run for about three days with one CPU-core.

**Fig 5:**
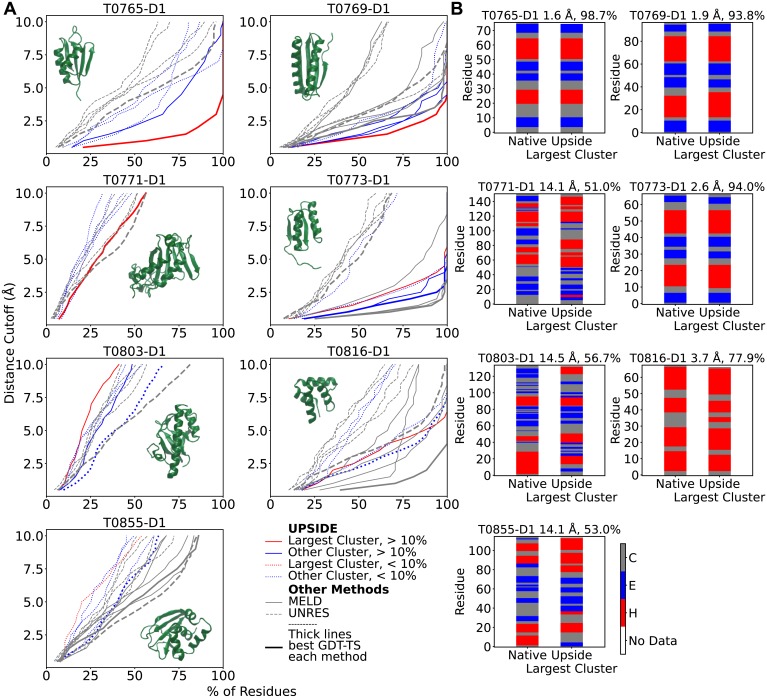
*Upside*, UNRES and MELD’s performance on seven CASP11 Targets. (A) Hubbard plots for the centroid of *Upside*’s top five clusters are compared to the UNRES’s and MELD’s five submitted structures. The length and the relevant residue range used in CASP11 analysis for each protein is shown along with the structure. (B) *Upside*’s secondary structure predictions for the centroid of the top cluster (C_*α*_-RMSD and secondary structure accuracy provided at top). The sequences provided by CASP11 organizers can be longer than the sequences used for evaluation due to disorder (e.g., for T0769-D1, simulations are conducted on 112 residues, but only the 97 folded residues are evaluated). The RMSD values provided are based on the CASP11-defined folded regions, and hence may differ slightly than those provided in Fig 4.

Contrastive divergence training has been shown to be effective for many machine learning problems [11], even without having simulations that converge to the Boltzmann ensemble. To test the accuracy of contrastive divergence on our protein model, we attempt *de novo* folding of a benchmark set of small, fast-folding proteins similar to those used in references [12–14] as well as various CASP11 targets investigated by other physics-based approaches (Figs 4 and 5) [15, 16]. Before training, we remove homologous proteins from the training set to help ensure that this would be a true *de novo* prediction.

Two temperature replica exchange simulations are run for each of the 23 proteins (14 replicas each). The first set is initialized from the native configuration to assess the stability of the experimental structure for the potential obtained from contrastive divergence training. The second set is initialized from an unfolded state (random Ramachandran *ϕ* and *ψ* angles) to test *Upside*’s capability to find the native structure which is reflection of both the accuracy of the energy function and the method’s ability to search conformational space. Each range of temperatures is chosen to be large enough to cover the unfolding transition for each given protein. We judge the accuracy and equilibration from the histograms of the C_*α*_-RMSD from the native structure after discarding the initial third of the simulation as equilibration (Fig 4).

The majority of the proteins show a small number of well-defined basins that represent the dominant conformations with the current potential. While the simulations often produce several conformations quickly, equilibration of their populations takes longer, on the order of CPU-days for some proteins, though still extremely short in comparison to typical molecular dynamics simulations.

For all 20 proteins below 100 residues, the lowest C_*α*_-RMSD structure obtained starting from an unfolded state is within 5 Å of the native state (54% within 3 Å). In some cases, the lowest C_*α*_-RMSD structure is in the largest cluster, while for other proteins, the best structure is in a minor cluster even when it is within 3 Å (e.g., gpW, NTL9). The designed 3-helix bundle, *α*3d [17], has a mirror image as a second heavily populated cluster.

When the native-initialized and unfolded-initialized structures have similar C_*α*_-RMSD distributions, the simulations are likely converged. Half of the proteins are approximately converged by this criterion (e.g., BBA, protein B, homeo domain, *α*3d and WW), but others are not, (e.g., protein L and ubiquitin). Convergence is achieved for a variety of proteins with the native or near-native structure being the dominant conformation (e.g., BBA, homeo domain, protein B). These proteins represent the ideal scenario in terms of both accuracy and convergence. But, convergence can be achieved even when the native conformation is not the dominant conformation (e.g., BBL, λ-repressor, NuG2). This result indicates that for these proteins, our energy function is inadequate in regards to identifying the native structure even though there is adequate sampling. For cspA, a relatively small protein having a complex all *β* fold, additional simulations run at constant temperature can find a stable structure having significantly lower C_*α*_-RMSD (3.6 rather than 6.1 Å); this finding points to the search process being the limiting factor rather than *Upside*’s energy function.

The *Upside* simulations tend to achieve the correct secondary structure with a small number of distinct tertiary arrangements. This diversity in tertiary structures occurs as mirrored three helix bundles for *α*3d and protein B, as well as the subtle re-arrangements of NuG2. For the three largest CASP11 targets we investigated (115–178 residues), the secondary structure performance is noticeable poorer, implying a strong coupling between secondary and tertiary structure formation for these larger systems (Fig 5).

### Comparison with other physics-based approaches

Simmerling and coworkers folded 17 sub93 residue proteins using GPUs to obtain a microsecond of simulation time per day with their pairwise Generalize Born (GB) model trained to reproduce Poisson–Boltzmann solvation along with their ff99SB force field [14]. Impressively, their replex protocol folded 16 of the 17 proteins to within 3 Å C_*α*_-RMSD although the top cluster was greater than 10Å for five of the six largest proteins. Over-all, the performance is very similar to *Upside*’s in that 1-3 Å C_*α*_-RMSD structures are achievable on most proteins but the structures are not always in the largest cluster.

For seven CASP11 targets between 65-178 residues, we compared *Upside* with two physics-based approaches that participated in CASP11 (Fig 5): the Cornell-Gdansk group’s coarse-grained united residue model “UNRES” [16] and MacCallum, Perez and Dill’s highly accelerated molecular simulation method “MELD” (Modeling Employing Limited Data), a Bayesian approach that utilizes physically-based heuristics combined with atomistic implicit solvent simulations [15]. It should be noted that both methods employ PsiPred, a secondary structure predictor employing evolutionary information [18]. In contrast, *Upside*’s secondary structures emerge during folding solely are a result of our energy function.

For T0765-D1, a 76 residue *α*/*β* protein, *Upside*’s major cluster contains the native fold (Fig 5). The performance is reflected in a low flat trace for the cluster centroid in the Hubbard plot of the Global Distance Test (GDT) versus sequence percentage. This performance is superior to all five of UNRES’s submissions (there were no MELD submissions). For T0769-D1, a 112 residue *α*/*β* protein, both *Upside* and MELD perform very well, with UNRES’s best submission being only slightly worse. For T0771-D1 and T0803-D1, 178 and 134 residue *α*/*β* proteins, respectively, neither *Upside* nor UNRES’s performance is very good (no MELD submissions). For T0773-D1, a 77 residue *α*/*β* protein, MELD performs extremely well while one of *Upside* structure also has the native fold. UNRES performance is much poorer. For T0816-D1, a 68 residue helical bundle, MELD performs astonishingly well while *Upside*’s and UNRES’s performances also are commendable. For T0855-D1, a 115 residue *α*/*β* protein, both MELD and UNRES perform similarly and better than *Upside*, but none succeed in finding the native fold. Generally, the three approaches are capable of folding proteins of up to 94 residues, but are challenged with larger proteins.

### Characterization of folding behavior

**Fig 6:**
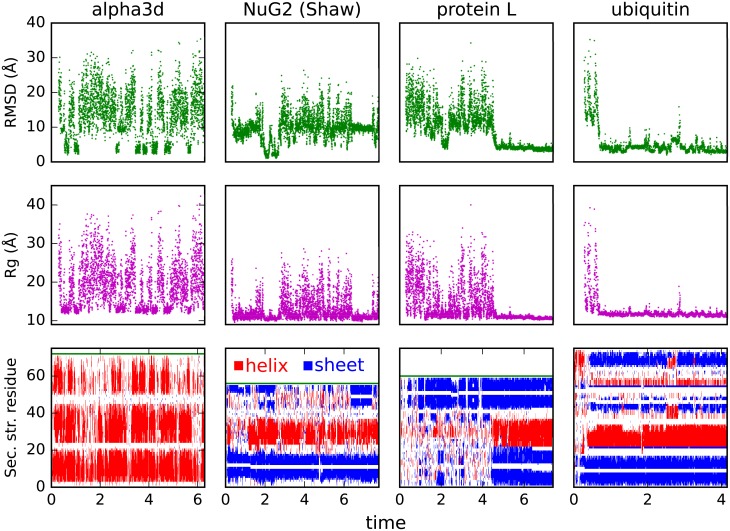
Constant temperature simulations. Trajectories are selected by the highest temperatures that still produce a significant population for the native state. Note that pivot Monte Carlo moves are attempted periodically which has little effect on folded dynamics but greatly decreases correlation time in the unfolded state.

**Fig 7:**
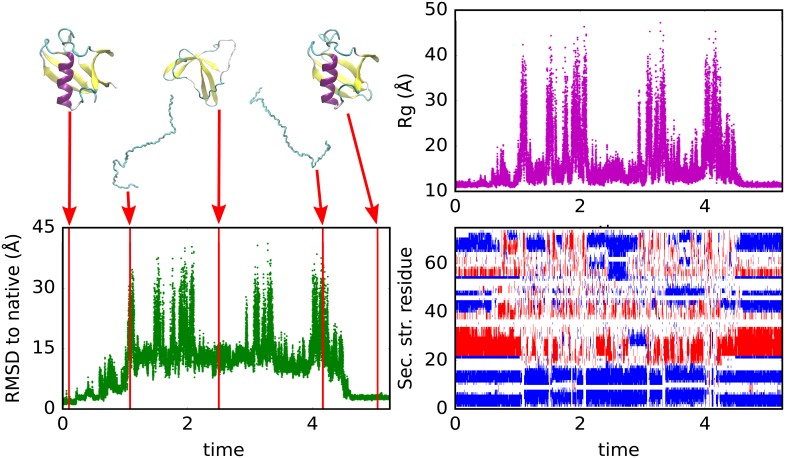
Constant temperature trajectory of ubiquitin. Simulation conducted at T = 1.00, initialized from the native structure, with representative structures along the trajectory highlighted. The 2nd and 4th structures are chosen for having a high *R*_g_ while the last structure is chosen based on minimum RMSD (2.3 Å.) after achieving full unfolding. Red and blue colors in lower right panel refer to helical and sheet secondary structures.

In constant temperature simulations, we observe reversible folding to the native state for a number of proteins in our test set in core-days (Figs 6 and 7). The time scales of folding indicated by these trajectories imply that the time scales we employed in the contrastive divergence simulations are far less (often a factor of 100 or more) than required to equilibrate these proteins, implying that contrastive divergence is optimizing only over fluctuations in or near the native well.

Note that conditional on low hydrogen bonding, the radius of gyration (*R*_g_) at high temperature and at the peak of the heat capacity are quite similar. This suggests the increase in *R*_g_ for the unfolded state as temperature increases is driven by a reduction in backbone-backbone hydrogen bonds rather than side chain effects.

Based on these results, two observations should be reconciled. The first observation is the presence of a sharp phase transition with a single peak for the heat capacity. The shape of the phase transition, but not its amplitude, is consistent with a cooperative folding transition. The second observation is the unrealistically large level of residual hydrogen bonding in the denatured state at temperature of the maximum in the heat capacity. Although the hydrogen bonding is less than that in the native state, the residual hydrogen bonding indicates that the transition is not fully cooperative. These observations may be explained by the essential feature of the contrastive divergence process, that it must balance the competing energy terms of the model so that no one energy dominates. More extensive training, for example using a more diverse ensembles that contain conformations outside the native well, may remove the excess hydrogen bonding.

**Fig 8:**
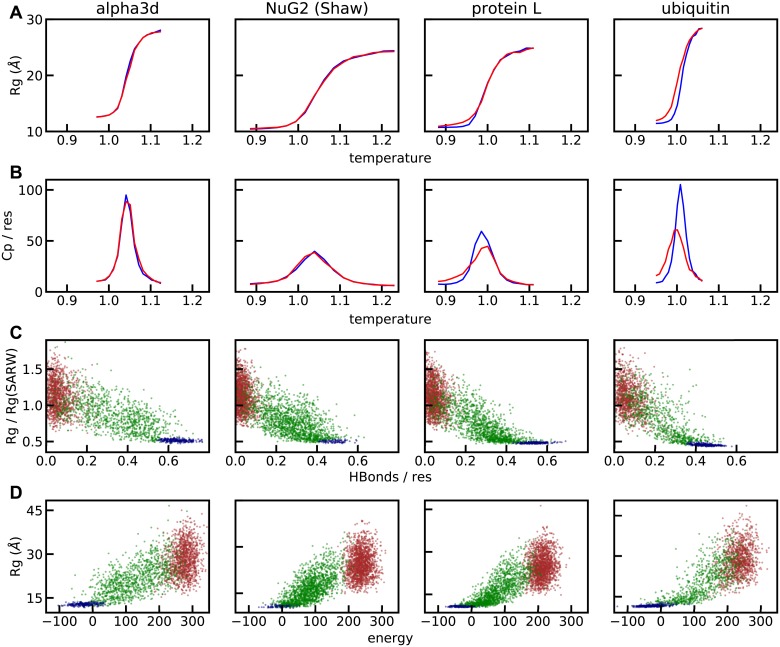
Thermodynamic behavior. The heat capacity is computed using the fluctuation relation *C*_p_ = (var *E*)/*T*^2^. The self-avoiding random walk *R*_g_ is computing using *R*_g_ = (1.9Å)(*N*_res_)^0.6^ for chemically-denatured proteins [19]. In the upper two panels (A) and (B), the *C*_p_ and *R*_g_ values are obtained from simulations started from either the native (blue line) or a random unfolded state (red line). In the lower two panels (C) and (D), the brown points are from high temperature simulations, while the green points (unfolded state) and blue points (folded state) are from simulations at the peak of the heat capacity. The simulation units are converted to physical units by assuming that the physical energy unit is 0.6 kcal/mol and that *T* = 1 corresponds to 300 K.

The *Upside* model exhibits concerted melting behavior over a small range of temperatures (Fig 8). While the temperature of the model in *Upside* is not exactly comparable to a physical temperature, it is reasonable to assume *T* = 1 corresponds roughly to a temperature of 300-310 K. The ubiquitin transition occurs over a temperature range of approximately 0.07 temperature units, or approximately a 20 K range, similar to that observed experimentally [20].

Furthermore, our temperature-denatured states have high *R*_g_ near the midpoint of the transition, consistent with experimental results and inconsistent with many all-atom molecular dynamics folding simulations [4, 21]. At the peak of the heat capacity, the *R*_g_ is ∼15% smaller than the predicted from experimental data while the *R*_g_ at high temperature is ∼10% larger than the experimental value. Both *R*_g_ values are significantly larger than those in most atomistic molecular dynamics simulations [4].

## Discussion

A major challenge in protein chemistry is to extract from a set of proteins the underlying interaction energies that capture the physiochemistry governing their folded structures adn dynamics. We addressed this challenge by showing that a strong connection exists between properties of the native basin and the rest of the protein’s conformational landscape, and this connection is strong enough to train a potential for *de novo* folding simulations. Furthermore, the resulting potential is inexpensive enough to equilibrate simulations of small proteins in CPU core-days on a commodity computer.

Specifically, we have developed a procedure involving extremely short simulations in the native energy well, coupled with optimization using contrastive divergence, to parameterize a sophisticated coarse-grain model. Underlying the model is a re-evaluation of the common assumption that increased detail is the path to greater accuracy. This requirement for detail is mitigated with trajectory-based training because less expensive models allow more extensive exploration leading to higher accuracy. We have also shown that very large numbers of parameters (even ∼20000 in our case) are no obstacle to producing accurate proteins models using trajectory-based training. While over-fitting is always a concern, the severity is greatly reduced because contrastive divergence is training *against* the vast possibilities of alternative protein conformations explored by conformational sampling. Additionally, contrastive divergence automatically obtains balanced parameters such that no particular interaction overwhelms the others. We contend that this balance between parameters is more important than the accuracy of any particular term.

Decoupling representations of protein physics is a key aspect of the *Upside* model. In particular, Upside decouples the representation of the protein used for dynamics, an N–C_*α*_–C backbone model, from the representation used for computing energies and forces, a complex representation that includes oriented side chain interactions. This combination allows us to build up the sophisticated coordinates needed to represent solvent exposure of side chains, geometry of hydrophobic packing, and side chain-backbone hydrogen bonding without the cost of running dynamical simulation on a complex model with slow equilibrium. The largest improvement comes from applying belief propagation to the side chain degrees of freedom so that we represent detailed side chain physics at the *χ*_1_/*χ*_2_-level without incurring the roughening of the energy landscape and slowing of the dynamics normally associated with detailed sterics of side chain interactions. It is an open question to determine how much molecular detail must be retained for accurate protein energetics, but *Upside* provides a flexible framework to explore these issues without compromising the simple backbone representation of dynamics.

### Related work

Contrastive divergence optimization has been applied to Gō-like protein potentials sampled with crankshaft Monte Carlo moves [22, 23]. These works optimized only tens of parameters, and the resulting model is used to fold protein G and 16-residue peptides.

Other studies have trained protein energy functions using libraries of decoys [24]. Such efforts are challenging because atomic energy functions have rugged energy landscapes where even small structural differences can produce large energy differences. This ruggedness implies that scoring decoys by energy without first relaxing them is problematic for the sharply-defined force fields necessary to describe protein physics, a problem that contrastive divergence avoids.

A distinction between contrastive divergence and traditional training methods, such as Z-score optimization [25], relates to the goal and the source of the decoys. In contrastive divergence, the critical task is to produce a high population of low RMSD structures with the model. Z-scoring training attempts to make the energy of the native state much lower than the average energy of an pre-constructed decoy library. This is problematic because the decoys may not have structures that exhibit the pathologies of a poorly-trained model. Additionally, we believe optimization should concentrate on the lowest energies that have significant Boltzmann probability, not the average energy which is dominated by highly-unlikely structures. Furthermore, it is difficult to evaluate the reliable energies of decoys without relaxing the decoys. Methods based on simulation ensembles (such as maximum likelihood and contrastive divergence) are well-defined and do not need pre-constructed decoy libraries.

Podtelezhnikov et al. [26] apply contrastive divergence to few-parameter protein models to optimize the parameters of hydrogen bond geometry. Their work is similar to this paper but narrower in scope.

The maximum likelihood method requires the computation of the derivative of the free energy, which involves a summation over an equilibrium ensemble. Such a requirement necessitates a very long simulation to update parameters. Still, this approach can be viable when used with very small proteins on which the simulations converge quickly. A variant of maximum likelihood is given in Ref. [27], where decoys are generated and a maximum likelihood model is fit to adjust the parameters to distinguish between near-native and far-from-native conformations. The potential is trained on a single protein, tryptophan cage, and then the resulting potential is applied to a number of *α*-helical proteins with some success.

### Time and temperature scale

The precise time scale and temperature scale of the *Upside* models is intentionally left arbitrary because the coarse-graining process may leave us without a linear relationship to physical time and temperature. The speed-up of *Upside* simulation due to the smoothing of side chain interactions is likely to have a disproportionate effect on time scales for condensed structures as compared to extended structures. Regardless, the equilibrium population distribution that determines the free energy is expected to be approximately correct, as well as the order of dynamical folding events. The precise relationship of *Upside* time scales to physical time scales is left to future work.

### Conclusion

By employing the computationally fast yet detailed *Upside* model, we can use multiple trajectories to train tens of thousands of parameters simultaneously to simulate protein folding and dynamics. The training successfully produces low-energy, native or near-native structures with sharp folding transitions for most of our validation proteins. The strategy’s success argues that simpler (in atomic representation) models that can be globally parameterized can rival more detailed but slower models whose parameterization is more challenging. We achieve success for some proteins in terms of accurately folding to low energy native state and achieve thermodynamic equilibration, but still fail on others. We hypothesize that the short-time contrastive divergence we are using does not provide a sufficient library of large changes in the tertiary structure to enable the potential to properly distinguish the various conformations. This issue will be addressed in future studies. Coupling large computational resources with Markov state models [28] should improve training of the *Upside* model by exploring a larger and more diverse conformational landscape on each contrastive divergence step.

The ready generation of Boltzmann ensembles allows for a wide range of computational studies of protein folding, dynamics, and binding. For example, computational screening of large numbers of proteins for foldability should be tractable as is the study of hydrogen exchange and folding kinetics. Additionally, in studies that incorporate experimental or bioinformatics data, including contact predictions, *Upside* provides an inexpensive Bayesian prior distribution over protein structures that may be updated using experimental information. This provides accurate predictions that make essential use of the totality of protein physics as encoded in the *Upside* model, while being inexpensive enough to allow validation and iteration on large numbers of proteins.

## Supporting information

S1 Text Derivation, model, and optimization details. (PDF)
