# Supplementary material for: Trajectory-based training enables protein simulations with accurate folding and Boltzmann ensembles in cpu-hours
Source: PLoS Comput Biol. 2018 Dec 27;14(12):e1006578. doi: 10.1371/journal.pcbi.1006578 (PMC6307714; doi:10.1371/journal.pcbi.1006578)
Supplement: S1 Text — (PDF) [file pcbi.1006578.s001.pdf]

Supporting Information (S1 Text)

Jumper et al.

Derivation of contrastive divergence

We derive the contrastive divergence method as a series of approximations to the problem of best approximating the probability distribution of observed PDB structures using a force field of an imperfect, fixed form. The initial part is a standard derivation of the maximum likelihood method, adapted to make clear its connection to protein molecular dynamics, while the end of the derivation makes clear the relaxation to obtain contrastive divergence as an approximation to maximum likelihood.

We begin by assuming that we have a large collection of protein sequences $\{s_a\}$ and their associated Boltzmann distributions $p_{s_a}^{\text{true}}(X_a)$ under physiological conditions, where a represents an arbitrary label to enumerate the proteins and X_a represents the configuration of the protein. Note that the "true" Boltzmann distribution is an unobservable idealization of the conformational ensemble of a protein under physiological conditions, and we further idealize that the true Boltzmann distribution is derived from from an extremely-complicated true potential $V_{s_a}^{\text{true}}$ by statistical mechanics,

$$p_{s_a}^{\text{true}}(X_a) = \frac{\exp(-V_{s_a}^{\text{true}}(X_a))}{\exp(-G_{s_a}^{\text{true}})} \tag{1}$$

$$G_{s_a}^{\text{true}} = -\log \int e^{-V_{s_a}^{\text{true}}(X)}\, dX. \tag{2}$$

The subscript s_a indicates that both the potential $V_{s_a}^{\text{true}}$ and free energy $G_{s_a}^{\text{true}}$ depend on the sequence of the protein. We may think of this as an artifact of working in the coarse-grained coordinates of the backbone trace, where the energy $V_{s_a}^{\text{true}}$ really represents the free energy of the backbone coordinates after integrating away the solvent and side chain degrees of freedom. An analogous situation occurs in parameterizing all-atom molecular dynamics, where the "energy" of the system really represents the free energy of the system after integrating over the electronic degrees of freedom. Our goal is to define a parametric $V_s^{\text{approx}}(X)$ that approximates the V_s^{true} for any sequence s. We drop the subscript s below where there is no possibility for confusion.

For an approximate potential V^{approx}, it is almost certain that V^{approx} does not have enough flexibility in its functional form to match all of the Boltzmann distributions p_a for any sequence s_a. We instead find a V^{approx} that is "close" to V^{approx}. Defining the Boltzmann distribution of V^{approx} in the same manner as that of V^{true},

$$p_{s_a}^{\text{approx}}(X_a) = \frac{\exp(-V_{s_a}^{\text{approx}}(X_a))}{\exp(-G_{s_a}^{\text{approx}})} \tag{3}$$

$$G_{s_a}^{\text{approx}} = -\log \int e^{-V_{s_a}^{\text{approx}}(X)}\, dX, \tag{4}$$

we may use the Kullback-Leibler (KL) divergence to measure the similarity of the associated Boltzmann

distributions,

$$
\begin{aligned}
&\mathrm{KL}(p^{\mathrm{true}}, p^{\mathrm{approx}}) \\
&\quad = \int p^{\mathrm{true}}(X) \log \frac{p^{\mathrm{true}}(X)}{p^{\mathrm{approx}}(X)}\, dX \\
&\quad = \langle -\log p^{\mathrm{approx}}(X) + \log p^{\mathrm{true}}(X) \rangle_{\mathrm{true}} \\
&\quad = \langle (V^{\mathrm{approx}}(X) - G^{\mathrm{approx}}) - \\
&\qquad (V^{\mathrm{true}}(X) - G^{\mathrm{true}}) \rangle_{\mathrm{true}}.
\end{aligned} \tag{5}
$$

In the last equation, we note that the KL divergence is simply the average energy difference between the true and approximate potentials (after subtracting the free energies to normalize the probabilities), where the average is taken over the true Boltzmann distribution. The key fact when minimizing KL divergence is that if the approximate distribution lacks the freedom to exactly match the true distribution, then the minimizing distribution will be weaker than the true distribution (i.e. less sharp) to avoid assigning highly unfavorable energy to configurations that are likely in the true distribution.

Dropping constant terms, we may instead minimize

$$
\langle V^{\mathrm{approx}}(X) - G^{\mathrm{approx}} \rangle_{\mathrm{true}}, \tag{6}
$$

since the remaining term $\langle V^{\mathrm{true}}(X_a) - G^{\mathrm{true}} \rangle_{\mathrm{true}}$ is independent of the approximating potential. This expectation value is still intractable since we do not know p^{true}, but we can approximate,

$$
p^{\mathrm{true}}(X) \approx p^{\mathrm{empirical}}(X) = \frac{1}{M} \sum_{a=1}^{M} \delta(X - X_a), \tag{7}
$$

where δ is the Dirac delta function and M is the number of proteins. This gives the objection function,

$$
\begin{aligned}
&\langle V^{\mathrm{approx}}(X) - G^{\mathrm{approx}} \rangle_{\mathrm{empirical}} \\
&\quad = \frac{1}{M} \sum_{a=1}^{M} \int \delta(X - X_a)(V^{\mathrm{approx}}(X) - G^{\mathrm{approx}})\, dX \\
&\quad = \frac{1}{M} \sum_{a=1}^{M} (V^{\mathrm{approx}}(X_a) - G^{\mathrm{approx}}).
\end{aligned} \tag{8}
$$

Minimizing the expression Eq 8 is exactly the method of maximum likelihood. The derivation given above illustrates two points via the connection to KL divergences. The first is that, if V^{approx} is insufficiently detailed, the model's ensemble will be overly broad to ensure no experimental conformation has high energy under V^{approx}. The second point is that with only a finite number of samples, $p^{\mathrm{empirical}}$ may be a poor approximation to p^{true}, which would allow V^{approx} to wrap itself tightly near the δ-functions associated with each sample. This is the origin of overfitting in maximum-likelihood models.

We can now take the derivative with respect to an arbitrary force field parameter α_i in preparation to perform gradient descent on Eq 8. The gradient is given by

$$
\begin{aligned}
&\frac{d}{d\alpha_i} \frac{1}{M} \sum_{a=1}^{M} (V^{\mathrm{approx}}(X_a) - G^{\mathrm{approx}}) \\
&\quad = \frac{1}{M} \sum_{a=1}^{M} \left(\frac{dV^{\mathrm{approx}}}{d\alpha_i}(X_a) - \frac{dG^{\mathrm{approx}}}{d\alpha_i} \right) \\
&\quad = \frac{1}{M} \sum_{a=1}^{M} \left(\frac{dV^{\mathrm{approx}}}{d\alpha_i}(X_a) - \left\langle \frac{dV^{\mathrm{approx}}}{d\alpha_i}(X) \right\rangle_{\mathrm{approx}} \right),
\end{aligned} \tag{9}
$$

where we have used the standard statistical mechanics identity $dG/d\alpha_i = \langle dV/d\alpha_i \rangle$. While we have obtained a concrete expression for gradient descent in Eq 9, we still have a major stumbling block. Computing the

expectation of the derivative of the potential at X_a is straightforward given a functional form for V^{approx}, but obtaining even a reliable approximation for $\left\langle \frac{dV^{\text{approx}}}{d\alpha_i}(X) \right\rangle_{\text{approx}}$ is extraordinarily difficult. To approximate the expectation value on a rough protein energy landscape, we would need Boltzmann samples from our current approximating potential. Even obtaining the single most likely configuration for our approximating potential is equivalent to finding the native state of the model, and this is very difficult for realistic pairwise potentials. Instead, we require the Boltzmann ensemble for all the proteins in our training set, and must update those Boltzmann ensembles as we use gradient descent to optimize the approximating potential. This represents an extreme expense and is unrealistic for anything but the simplest models of proteins. Note also that we cannot simply construct a large list of structures at some time and reweight those structures according to the potential, since the potential is constantly changing. Reweighting ensembles is only valid over very small neighborhoods of parameter space, and this procedure would depend on being able to generate an exhaustive survey of candidate structures in an exponentially large space.

The contrastive divergence method [1] approximates the maximum likelihood procedure using an empirical observation. We do not need an accurate approximation to Eq 9, so long as the derivative points in direction of parameter space that improves the potential accuracy (i.e. any direction is acceptable as long as it is not uphill). Hinton proposes replacing Boltzmann average $\left\langle \frac{dV^{\text{approx}}}{d\alpha_i}(X) \right\rangle_{\text{approx}}$ with a finite-time Fokker-Planck average over a very short period of time *for a simulation that originates at the data point X_a*. In the Monte Carlo (MC) dynamics that the original authors use, even one MC step is sufficient to produce acceptable optimization of their model. In our case, we replace their small number of MC steps with a short time simulation using replica exchange Langevin dynamics. As the duration of the simulation is increased, our derivative estimate will converge to the true derivative Eq 9. Our paper empirically demonstrates that equilibrating each model within only a local region around the crystallographic native state is sufficient for a good folding model, so long as a large and diverse collection of protein structures are jointly optimized.

Optimization and simulation details

The following temperature ranges are used for the replica exchange simulations with 14-16 replicas per simulation. These temperatures are chosen to use the minimal temperature range that approximately span the thermal melting transition for each protein using information from an earlier set of replica exchange simulations. The temperatures of the simulation initialized from the crystal structure and those initialized from extended structures use the same temperature range.

The force is integrated using Verlet integration with a time step of 0.009 time units. Temperature is maintained using a Langevin thermostat with a thermalization timescale of 0.135 time units.

Simulation times in all figures are given in millions of *Upside* time units (approximately 10^8 force evaluations).

Handling crystallographic artifacts

The derivation of contrastive divergence makes the assumption that the conformations X_a are equilibrium samples from the Boltzmann distribution of each protein, but in reality, we must work with crystal structures of proteins. It has been shown that that the static diversity of crystal structures for different proteins conveys significant information about the dynamic ensembles of individual proteins [3]. Crystal structures deviate in a number of systematic ways from equilibrium samples, but we are most concerned about crystal packing artifacts, crystallizability bias, and errors in published structures.

We expect that our bias in working only with crystallizable sequences, thus missing intrinsically disordered regions from training, likely biases the resulting potential to disfavor coil states. The loop-stabilizing effects of crystal packing somewhat counteract this effect, as it allows longer loop regions to exist in crystal structures.

Training data and optimization

The contrastive divergence training is conducted with 456 crystal structures from the Protein Data Bank. The initial selection of structures uses the PISCES server [4] to select proteins with X-ray resolution less than 2.2 Å and pairwise sequence similarity less than 30%. In structures with multiple chains, a single chain is

Table A: Temperatures for replica exchange

Protein	T_{min}	T_{max}
alpha3d	0.972	1.123
BBA	0.757	1.499
BBL	0.844	1.202
cspA	0.925	0.975
gpW	0.850	1.000
homeodomain	0.978	1.153
hyp	1.000	1.050
lambda	0.879	1.172
NTL9	0.776	1.139
NuG2 (Shaw)	0.975	1.050
protein B	0.885	1.172
protein G	0.776	1.295
protein L	0.883	1.109
top7	1.090	1.130
ubiquitin	0.951	1.060
WW domain	0.704	1.295
T0765	0.970	1.000
T0769	0.940	0.980
T0771	0.975	1.050
T0773	0.850	0.980
T0803	0.920	1.020
T0816	0.980	1.020
T0855	0.980	1.050

chosen by the PISCES server. To avoid non-globular proteins or proteins with strong interactions with other subunits in the structure, random sample consensus linear regression [5] is used to identify outliers based on the relationship between $\log N_{\mathrm{res}}$ and $\log R_g$. Only chains with between 50 and 100 residues are used to encourage fast relaxation during the contrastive divergence simulations. All proteins homologous to proteins in the benchmark folding set are eliminated from the training set. Additionally, all proteins with backbone gaps, either missing residues due to diffuse electron density or non-standard amino acids that *Upside* does not handle, are also excluded from the training set.

The final training set of 456 proteins is divided into 38 groups of 12 proteins each, called minibatches. The Adam optimizer, a popular machine learning algorithm [6] is used to perform gradient descent on the objective function, using the contrastive divergence pseudo-gradient in place of the true maximum likelihood gradient. The Adam parameters used are $\beta_1 = 0.8$, $\beta_2 = 0.96$ and $\epsilon = 10^{-6}$. The α parameter is varied based on the type of term to ensure stability, $\alpha_{\mathrm{SC}} = 0.5$, $\alpha_{\mathrm{env}} = 0.1$, $\alpha_{\mathrm{HBond}} = 0.02$, and $\alpha_{\mathrm{sheet}} = 0.03$. The α parameters are multiplied by 0.25 for the fine-tuning optimization.

Regularization and derivative propagation for contrastive divergence optimization are handled using the Theano python library for automatic differentiation [7].

Burial definition

To handle the uncertainty of rotameric states that can affect the count, the count for different rotameric states are weighted by the prior probabilities of the rotamer states, given by

$$N_i = \sum_{\substack{j \\ |i-j|>2}} \sum_{\chi_i} p(\chi_i) S(|y_i(\chi_i) - y_i^{\mathrm{C}_\beta}| - (8\,\text{Å}), (1\,\text{Å})) S(\mathrm{angle}(y_i(\chi_i) - y_i^{\mathrm{C}_\beta}, d_i^{\mathrm{C}_\beta}) + 0.1, 1), \quad (10)$$

where S is sigmoid-like cutoff function, y^{C_β} is the position of the C_β, and d^{C_β} is the C_α–C_β bond direction.

Table B: Protein sequences and structures

Name	PDB ID	Length	Sequence
alpha3d	2a3d	73	MGSWAEFKQRLAAIKTRLQALGGSEAELAAFEKEIAA FESELQAYKGKGNPEVEALRKEAAAIRDELQAYRHN
BBA	1fme	28	EQYTAKYKGRTFRNEKELRDFIEKFKGR
BBL	2wxc	47	GSQNNDALSPAIRRLLAEWNLDASAIKGTGVGGRLTREDVEKHLAKA
cspA	1mjc	69	SGKMTGIVKWFNADKGFGFITPDDGSKDVFVHFSAIQND GYKSLDEGQKVSFTIESGAKGPAAGNVTSL
gpW	2l6q	62	MVRQEELAAARAALHDLMTGKRVATVQKDGRRVEFTATSV SDLKKYIAELEVQTGMTQRRRG
homeodomain	2p6j	52	MKQWSENVEEKLKEFVKRHQRITQEELHQYAQ RLGLNEEAIRQFFEEFEQRK
hyp	1whz	70	MWMPPRPEEVARKLRRLGFVERMAKGGHRLYTHPDGRIVV VPFHSGELPKGTFKRILRDAGLTEEEFHNL
lambda	1lmb	80	PLTQEQLEDARRLKAIYEKKKNELGLSQESVADKMGMGQS GVGALFNGINALNAYNAALLAKILKVSVEEFSPSIAREIY
NTL9	2hba	39	MKVIFLKDVKGMGKKGEIKNVADGYANNFLFKQGLAIEA
NuG2 (Shaw)	1mi0	57	MDTYKLVIVLNGTTFTYTTEAVDAATAEKVFKQY ANDAGVDGEWTYDAATKTFTVTE
protein B	1prb	53	TIDQWLLKNAKEDAIAELKKAGITSDFYFNAINKAKTVEEVNALKNEILKAHA
protein G	1pga	56	MTYKLILNGKTLKGETTTEAVDAAT AEKVFKQYANDNGVDGEWTYDDATKTFTVTE
protein L	2ptl	61	VTIKANLIFANGSTQTAEFKGTFEKATSE AYAYADTLKKDNGEYTVDVADKGYTLNIKFAG
top7	1qys	92	DIQVQVNIDDNGKNFDYTYTVTTESELQKVLNELMDYIKKQGAKRV RISITARTKKEAEKFAAILIKVFAELGYNDINVTFDGDTVTVEGQL
ubiquitin	1ubq	76	MQIFVKTLTGKTITLEVEPSDTIENVKAKIQDKEGIPP DQQRLIFAGKQLEDGRTLSDYNIQKESTLHLVLRLRGG
WW domain	2f21	33	KLPPGWEKRMSADGRVYYFNHITNASQWERPSG
T0765		76	QQQEATLAIRPVGQGIGMPDGFSVWHHLDANGIRFKSIT PQKDGLLIKFDSTAQGAAAKEVLGRALPHGYIIALLE
T0769		112	MLTVEVEVKITADDENKAEEIVKRVIDEVEREVQKQYP NATITRTLTRDDGTVELRIKVKADTEEKAKSIIKLIEE RIEEELRKRDPNATITRTVRTEVGSSWSLEHHHHHH
T0771		178	TEESLEGTVIYKKTTTFEVDGYTYQCDVDDGSQFVTLYNK ENKLTYEKIVYKDTGKTYIGSWSSNVIEYDRFMSQQADFI VDQAFTKAMADEIGKTELMITMLLSPNTGEVMEVNFNFFT EPYAKVPLHVYREIEVKLKEQIHFKPIEEGKQLNYIMLA WMQKPQGKLPPLPPPGSL
T0773		77	MVDLKIDVSDDEEAEKIIREIREQWPKATVTRTNGDIKLD AQTEKEAEKMEKAVKKVKPNATIRKTGGSLEHHHHHH
T0803		134	SNINKAKVASVESDYSSIKSAALSYYSDTNKIPVTPDGQT GLNVLETYMESLPDKADIGGEYKLIKVGNKLVLQIGKDGE GVTLTEAQSAKLLSDIGKDKIYTGVTGDNFGEQLKDTTKI DNKALYIVLIDNTV
T0816		68	MITYKKLLDELKKEIGPIAKIFLNKAMESLGYDDVDDSNYK EILSVLKMNKELREYVEIVEERLEKEG
T0855		115	FTDQQIGVLAGLAISPEWLKQNIAANQLVYGIVKPSDTVP AGVDDYSYLVAADDQDGTIIFFKAEGQTVIIKYTSQRNTK LKAKALTLSQLKKEFYQTRSQKREVDDYVAGLRTE

Mutations from the listed PDB structures are indicated in red. The NuG2 sequence is from reference [2].

Other potential terms

The hydrogen bonding interaction is computed from the distance between hydrogen and oxygen r_{HO}, the separation vector d_{HO} as well as the hydrogen and oxygen bond vectors d_{H} and d_{O}, respectively. The strength of a single interaction is given by

$$h = f(r_{\mathrm{HO}})g(d_{\mathrm{HO}} \cdot d_{\mathrm{O}})g(-d_{\mathrm{HO}} \cdot d_{\mathrm{HO}}) \tag{11}$$

$$f(r) = \mathrm{sigmoid}\left(\frac{r - 1.4\,\text{Å}}{0.1\,\text{Å}}\right) \mathrm{sigmoid}\left(\frac{2.5\,\text{Å} - r}{0.125\,\text{Å}}\right) \tag{12}$$

$$g(c) = \mathrm{sigmoid}\left(\frac{c - 0.682}{0.05\,\text{Å}}\right). \tag{13}$$

The interactions are combined to ensure that each hydrogen bond can have at most one full partner using

$$V_{\mathrm{hbond}} = E_{\mathrm{hbond}} \sum_i \left(2 - \prod_{j \neq i}(1 - h_{ij}) - \prod_{j \neq i}(1 - h_{ji})\right), \tag{14}$$

which is inspired by treating each h_{ij} as the probability that residue i donates a hydrogen bond to residue j. The energy is then linear in the probability has at least one hydrogen bond donor and linear in the probability it has at least one hydrogen bond acceptor. The parameter E_{hbond} is learned by contrastive divergence.

The backbone steric interactions are imposed with approximately 3 Åsoft sphere repulsion among the N, C^{α}, C, and C^{β} (placed) atoms.

References

1. Hinton GE. Training products of experts by minimizing contrastive divergence. Neural computation. 2002;14(8):1771–1800.

2. Lindorff-Larsen K, Piana S, Dror RO, Shaw DE. How fast-folding proteins fold. Science. 2011;334(6055):517–520.

3. Jha AK, Colubri A, Freed KF, Sosnick TR. Statistical coil model of the unfolded state: resolving the reconciliation problem. Proceedings of the National Academy of Sciences of the United States of America. 2005;102(37):13099–13104.

4. Wang G, Dunbrack RL. PISCES: a protein sequence culling server. Bioinformatics. 2003;19(12):1589–1591.

5. Fischler MA, Bolles RC. Random sample consensus: a paradigm for model fitting with applications to image analysis and automated cartography. Communications of the ACM. 1981;24(6):381–395.

6. Kingma D, Ba J. Adam: A method for stochastic optimization. arXiv preprint arXiv:14126980. 2014;.

7. Theano Development Team. Theano: A Python framework for fast computation of mathematical expressions. arXiv e-prints. 2016;abs/1605.02688.
